# Gene Therapy Approaches to Functional Cure and Protection of Hematopoietic Potential in HIV Infection

**DOI:** 10.3390/pharmaceutics11030114

**Published:** 2019-03-11

**Authors:** Tetsuo Tsukamoto

**Affiliations:** Department of Immunology, Kindai University Faculty of Medicine, Osaka 5898511, Japan; ttsukamoto@med.kindai.ac.jp; Tel.: +81-723-66-0221

**Keywords:** human immunodeficiency virus, acquired immunodeficiency syndrome, hematopoietic stem/progenitor cells, gene therapy

## Abstract

Although current antiretroviral drug therapy can suppress the replication of human immunodeficiency virus (HIV), a lifelong prescription is necessary to avoid viral rebound. The problem of persistent and ineradicable viral reservoirs in HIV-infected people continues to be a global threat. In addition, some HIV-infected patients do not experience sufficient T-cell immune restoration despite being aviremic during treatment. This is likely due to altered hematopoietic potential. To achieve the global eradication of HIV disease, a cure is needed. To this end, tremendous efforts have been made in the field of anti-HIV gene therapy. This review will discuss the concepts of HIV cure and relative viral attenuation and provide an overview of various gene therapy approaches aimed at a complete or functional HIV cure and protection of hematopoietic functions.

## 1. Introduction

Human immunodeficiency virus (HIV) infects CD4^+^ T cells and causes acquired immunodeficiency syndrome (AIDS). AIDS remains as a global threat due to multifactorial reasons, including the difficulty in developing an effective vaccine [1]. According to The Joint United Nations Programme on HIV/AIDS (UNAIDS), in 2017, about 36.9 million people were living with AIDS while only 21.7 million patients were receiving antiretroviral therapy (ART), resulting in about 1.8 million newly HIV-infected people per year [2]. Although ART can limit the size and distribution of HIV reservoirs depending on the earliness of its initiation, it cannot eliminate latent HIV infections from the host and thus, a lifelong prescription is required for suppressing viral rebound from the reservoirs [3]. Therefore, further exploration is vital to discover new treatment options and effective vaccines [4].

**Figure 1:**
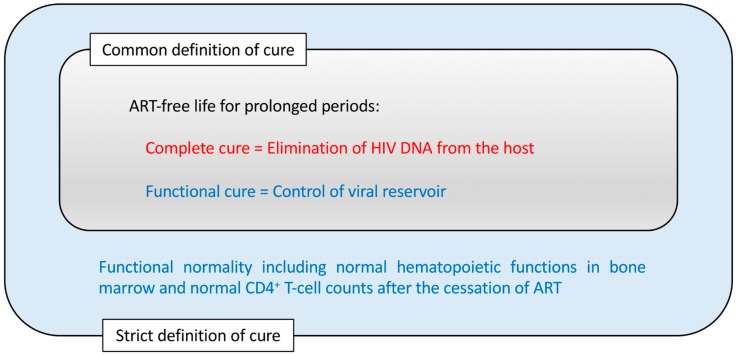
The concepts of human immunodeficiency virus (HIV) cure. A cure for the HIV disease is commonly interpreted as antiretroviral drug therapy (ART)-free life without viral rebound for prolonged periods. In addition, the cure for bone marrow dysfunctionalities observed in HIV-infected patients could be included in a stricter definition of a HIV cure.

The depletion of memory CD4^+^ T cells preceding AIDS manifestation may be mainly due to the infection of these cells. However, HIV may also reduce the production of naïve T cells by infecting CD4^+^ thymocytes [5,6,7,8]. On the other hand, although the dynamics of hematopoietic stem/progenitor cells (HSPCs) in HIV-infected settings is still unclear, it is well established that HIV infections are associated with hematological changes, such as anemia and pancytopenia [9]. These hematological changes are likely due to the modified HSPCs and hematopoietic potential of the host. Therefore, a cure for HIV disease should consider not only the absence of newly HIV-infected CD4^+^ cells but also the normal production rates of CD4^+^ T cells and other hematopoietic cells. To achieve an HIV cure in its strict sense, the protection of bone marrow hematopoietic functions is essential (Figure 1).

This review first describes the current evidence of modified bone marrow hematopoietic potential in HIV infection, leading to the strict definition of an HIV cure. It then explains how anti-HIV gene therapy methods applied to HSPCs can support the preservation of hematopoietic potential and a functional cure. This will be followed by an overview of different potential gene therapy methods for achieving this goal.

## 2. Evidence of Modified CD34^+^ Cell Dynamics and Functions in HIV Infection

HIV-1 may cause the loss of primitive hematopoietic progenitors without directly infecting these cells [10]. However, HIV infection does not cause the complete loss of CD34^+^ stem cells and therefore, it is possible to harvest stem cells from HIV-infected patients suffering from lymphoma [11] albeit with reduced efficiencies in relation to the reduction of peripheral CD4^+^ T-cell counts [12] or reduced in vitro lymphopoiesis capacities [13]. The recovery of CD4^+^ T-cell counts after successful antiretroviral drug therapy treatment may depend on the recovery of CD34^+^ cell counts [14].

A number of potential mechanisms have been suggested for the changes of CD34^+^ cells in the presence of HIV, such as reduced expression of the proto-oncogene *c-mpl* on CD34^+^ cells [15] and elevated plasma stromal cell-derived factor 1 (SDF-1) levels [16]. HIV-1 infection causes the upregulation of inflammatory cytokine production that may affect the dynamics and functions [17] or induce Fas-mediated apoptosis [18] of bone marrow CD34^+^ cells. On the other hand, HSPCs themselves may contribute to inflammation and allergies [19]. This may be partly due to the fact that inflammatory signals are involved in HSPC development [20]. Recent evidence has suggested that CD34^+^CD226(DNAM-1)^bright^CXCR4^+^ cells may represent a subset of common lymphoid progenitors associated with chronic HIV infection and inflammation, reflecting the altered dynamics of natural killer cells and α/β T cells [21].

Humanized mouse models are useful for analyzing bone marrow CD34^+^ loss or changes after the HIV-1 challenge. In studies with humanized mice infected with CXCR4-tropic HIV-1_NL4-3_, CD34^+^ hematopoietic progenitor cells were depleted and showed impaired ex vivo myeloid/erythroid colony forming capacities after the challenge [22,23]. A reduction of bone marrow CD34^+^ cell counts after CCR5-tropic HIV-1 infection was also detected in another study [24]. Interestingly, the depletion of bone marrow CD34^+^ cells following CCR5-tropic HIV infection has been reported to depend on plasmacytoid dendritic cells [25] or to be associated with the expression of CXCR4 [26]. The latter implicates a potential role of the SDF-1/CXCR4 axis in the loss of CD34^+^ cells. Another recent in vitro study suggested that CD34^+^CD7^+^CXCR4^+^ lymphoid progenitor cells may be depleted in the presence of CXCR4-tropic HIV-1 in the coculture of HIV-infected cord-derived CD34^+^ cells with mouse stromal OP9-DL1 cells, which allow the differentiation of T cells [27].

## 3. The Idea of Intracellular Immunization of HSPCs to Replace the Whole Hematopoietic System

After this, it is important to consider how we could deal with hematopoietic changes in HIV infection. A potential solution is gene therapy. In 1988, David Baltimore presented his idea of intracellular immunization by gene therapy [28] and his concepts are still valid today. First, he suggested expressing inhibitory molecules against HIV in target cells. Second, he proposed using retroviral vectors to transduce cells although lentiviral vectors are widely used today. Third, he conceived the use of gene-modified HSPCs to replace the immune system of the hosts with an HIV-resistant one. These concepts may be summarized as intracellular artificial immune systems designed against HIV and working independently from HIV-specific CD4^+^ helper T cells, which are the most vulnerable HIV targets [29]. Since his work, a number of candidate gene therapies have been proposed and tested and are described later in this article.

## 4. The Protection of Bone Marrow CD34^+^ Cells by an Anti-HIV Gene Therapy Demonstrated In Vivo

However, there have been few reports so far that have tested the protection of CD34^+^ cells after HIV infection by gene therapy. This may be because viral suppression and CD4^+^ counts have been widely accepted as measures for the effect of gene therapies against HIV. However, the true goal for any gene therapy against HIV should be the protection of hematopoietic potential because this is another arm of the definition of AIDS, i.e., the loss of cellular immunity (Figure 1).

**Figure 2:**
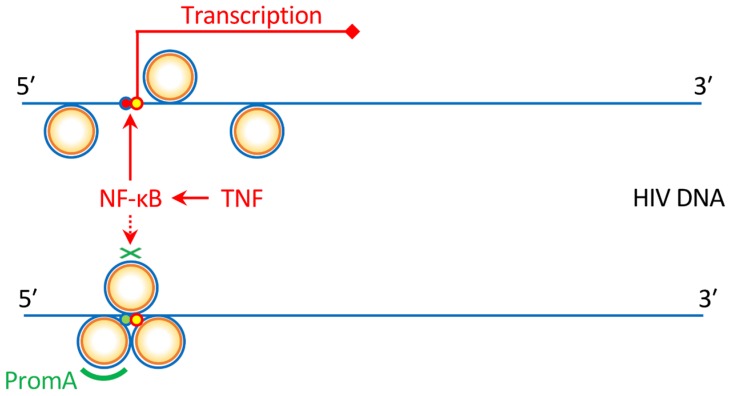
A schematic overview of PromA. PromA induces chromatin compaction in the human immunodeficiency virus (HIV)-1 promoter. This prevents HIV-1 DNA from reactivation, such as NF-κB-mediated reactivation by tissue necrosis factor (TNF). For details on the molecular mechanisms involved in transcriptional gene silencing induced by PromA, see Klemm et al., 2016 [30] and Mendez et al., 2018 [31].

**Figure 3:**
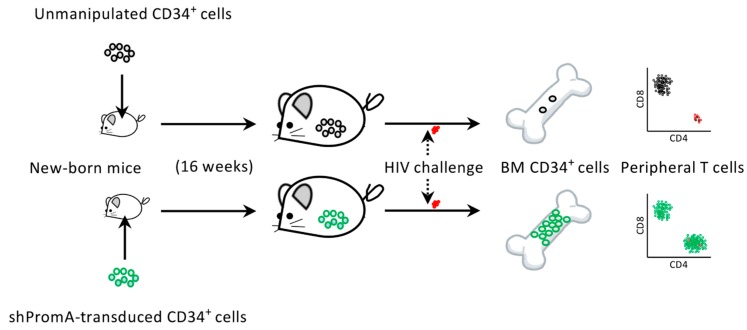
Summary of the humanized mouse study to test the efficacy of shRNA PromA (shPromA) [26]. Newborn NOD/SCID/Jak3^null^ mice were intrahepatically transfused with unmanipulated cord-derived CD34^+^ cells or CD34^+^ cells lentivirally transduced with shPromA. Those mice showing engraftment of human cells were challenged with CCR5-tropic HIV-1_JRFL_. Two weeks after the challenge, the mice were sacrificed and their bone marrow (BM) CD34^+^ cells and peripheral T cells were analyzed. Interestingly, mice transplanted with unmanipulated CD34^+^ cells showed unexpectedly low BM CD34^+^ cell counts 2 weeks after HIV infection, with concomitant depletion of peripheral CD4^+^ T cells. On the other hand, mice engrafted with shPromA-expressing CD34^+^ cells showed preserved BM CD34^+^ cell and peripheral CD4^+^ T-cell populations at 2 weeks post challenge.

Regarding this, we have recently reported that a transcriptional gene silencing (TGS) approach using a short hairpin (sh) RNA, which is called shPromA (Figure 2), resulted in limited CXCR4-associated depletion of bone marrow CD34^+^ cells following CCR5-tropic HIV infection in humanized mice (Figure 3). This suggests that anti-HIV gene therapy can support the preservation of the hematopoietic potential of the hosts [26]. Further characteristics of shPromA and previous studies testing its efficacy as a functional cure gene therapy method is discussed in Section 8.

## 5. Target Cells for Anti-HIV Gene Therapies

Recent studies, including the above shPromA study, indicate that ideal anti-HIV gene therapy targets should be hematopoietic stem cells rather than more differentiated cells, such as peripheral CD4^+^ T cells, because the transduced cells could engraft the host bone marrow and act as a lifelong source of HIV-resistant CD4^+^ cells [26,32,33]. Potential gene therapies using CD34^+^ cells have been investigated in vitro using cell culture experiments [26,34,35,36] or in vivo using humanized mice [26,35,37,38,39,40]. Furthermore, the transplantation of macaques with gene-modified autologous CD34^+^ cells followed by an infection with SIV has also been tested [41,42] although strategies may differ between gene therapies [33]. Based on such basic study results, the clinical trials using the transplantation of retrovirally or lentivirally gene-modified CD34^+^ cells in HIV-positive patients have been carried out [43,44,45]. Gene therapies of CD34^+^ cells have been considered as a cure for monogenic immune diseases. For example, the patients with adenosine deaminase deficiency [46], Wiskott–Aldrich syndrome (WAS) [47] and X-linked severe combined immunodeficiency [48,49] were successfully treated in clinical trials by transplantation of autologous CD34^+^ cells retrovirally or lentivirally transduced with the wild-type gene. Lentiviral vectors may be more efficient in gene transfer into resting stem cells at the G0/G1 phase compared with murine retroviral vectors [50]. If applied to the gene therapy of HSPCs, both retroviral and lentiviral vectors could have adverse effects, including the deregulation of gene expression [51] and the triggering of the p53 protein [52]. However, lentiviral vectors may be safer than retroviral vectors because the latter may occasionally cause insertional mutagenesis near the active start regions of genes, which could possibly lead to oncogenesis and cancers, such as leukemias [48]. Self-inactivating retroviral or lentiviral vectors lacking the U3 region of 3′ LTRs have further safety advantages [53]. Moreover, recent evidence has shown that the transplantation of WAS patients with autologous CD34^+^ cells transduced with lentiviral vectors encoding WAS protein results in the long-term survival of genetically engineered hematopoietic stem cells and lymphoid-committed progenitors [54]. Thus, this provides hope for lifelong protection from HIV.

Induced pluripotent stem cells (iPSCs) may also be candidates for anti-HIV gene transfer. iPSCs can be generated from the somatic cells of patients, which can differentiate to any cells in vitro and are expected to be utilized for the treatment of a broad range of genetic diseases [55,56,57,58]. Although CD34^+^ cells can engraft in the bone marrow following transplantation and differentiate to hematopoietic cells in vivo, iPSCs may be more convenient for in vitro hematopoiesis compared to CD34^+^ cells because of their ease of culture [59]. Interestingly, the impact of shPromA-transduced iPSCs on the suppression of viral replication in vitro has recently been demonstrated, suggesting that the large-scale production of gene-modified monocytes or lymphocytes in vitro for adoptive therapy could be a future option [60]. Additionally, the generation of iPSCs from HIV epitope-specific CD8^+^ cytotoxic T cells followed by their redifferentiation into the identical epitope-specific CD8^+^ T cells for adoptive transfer could be an effective immunotherapy [61].

## 6. Complete Cure vs. Functional Cure for HIV Infection

Before describing individual anti-HIV gene therapy methods, this review looks back on Figure 1 to summarize two major strategies for the treatment of HIV infection. One is to eliminate all the HIV DNA copies within the host, which is termed a complete cure (Figure 1). In pursuing the feasibility of this goal, tremendous efforts have been made to (1) find a method to detect all the latently infected HIV DNAs in viral reservoirs and to (2) eliminate all the detected HIV DNAs so that the host would become sterile in terms of HIV infection [62]. Among the methods to achieve this, the so-called “shock and kill” method, in which the reactivation of the viral reservoir is attempted with a shock-inducing agent followed by the immune-mediated killing of the reactivated cells, has been widely investigated [63,64,65,66,67]. These efforts have been partly successful [62,68]. However, the difficulty of viral eradication in vivo is not limited to HIV but include other viruses that induce long-lasting latent infections, such as herpes simplex viruses, varicella–zoster virus, cytomegalovirus and Epstein–Barr virus, making them ineradicable [69]. HIV may differ from other latently infecting viruses as the viral replication from the latent reservoir can resume quickly even if the host is not considered to be immunocompromised [70]. Moreover, even in the case of the Berlin patient who exhibited no sign of HIV existence following allogeneic transplantation with CCR5-Δ32/Δ32 hematopoietic stem cells, a complete cure was assumed rather than being fully demonstrated [71,72].

Alternatively, some potential gene therapy methods aim at a functional cure that is evidenced by the control of HIV replication below the limit of detection and the immune system being functionally normal despite residual cells harboring HIV proviral DNAs in the host (Figure 1) [68,73]. This approach might be more practical than the complete cure approach, given that many successful vaccines for chronic viral infections so far exert a functional cure rather than achieving the elimination of the targeted viruses [74]. In light of this, it could be stated that for those pathogens where an effective vaccine has not been developed to date, researchers could instead develop gene therapies aimed at a functional cure. In this way, there is an overlap between the concept of functional-cure gene therapy and the concept of vaccines against chronic pathogens [75]. In the next paragraph, the relevance of this is better clarified by looking at a similarity between live-attenuated vaccines and functional-cure gene therapy.

## 7. Connection between Functional-Cure Gene Therapies and Live-Attenuated Vaccine Approaches

**Figure 4:**
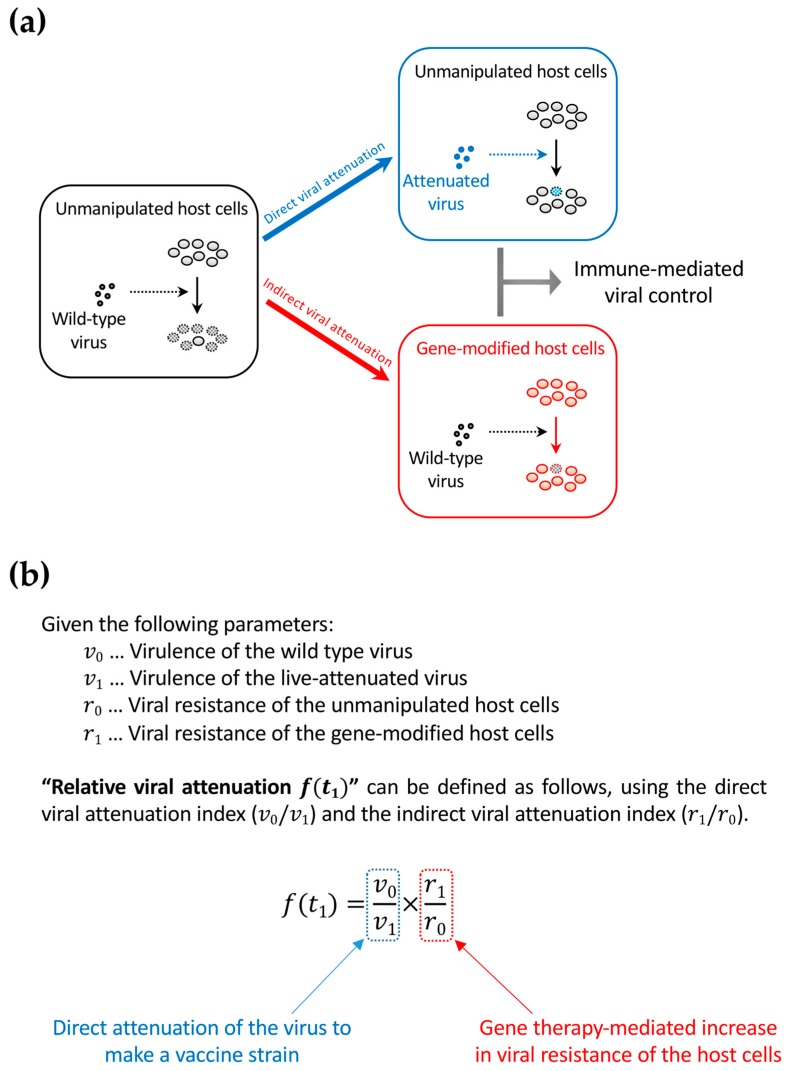
The concept of relative viral attenuation. (**a**) A schema describing direct and indirect viral attenuation. HIV usually infects host CD4^+^ cells efficiently and replicates rapidly. As a result, the host immune system fails to control viral replication (left). However, accumulating evidence in macaque AIDS models suggests that a live-attenuated virus, which infects and replicates slowly because of partial defects in the viral genome, can be controlled by the immune system and helps in further immunization against a potential superinfection with immunodeficiency virus strains that are homologous to the vaccine strain (upper right). The live attenuation method cannot be directly applied to HIV infection in humans because of safety concerns. However, indirect viral attenuation can be achieved by rendering the host cells HIV-resistant by an “intracellular immunization” gene therapy (lower right); (**b**) The definition of relative viral attenuation. This idea can connect live-attenuated vaccine studies and gene therapy approaches to achieve a functional cure. While live-attenuated HIV vaccines are not testable in humans for safety concerns, functional-cure gene therapy could be an alternative.

Anti-HIV gene therapy might be compared to some of the vaccine candidates tested so far in order to better foresee its future direction. Live-attenuated vaccines have been tested in macaque AIDS models using simian immunodeficiency virus (SIV) strains [76,77,78,79,80,81,82]. After the infection of a host with a live-attenuated SIV or simian-human immunodeficiency virus (SHIV), the vaccine strain is controlled by T-cell response but remains slowly replicating in the infected host. This results in further immunization of the host to prepare for the subsequent superinfections of wild-type SIV or SHIV. Therefore, even if live-attenuated vaccines are powerful, they provide a functional but not a complete cure. This means that there is a scientific connection between live-attenuated vaccines and gene therapy approaches for a functional cure because the latter confer viral attenuation indirectly by rendering the host cells HIV-resistant (Figure 4a). The two distinct strategies can be collectively interpreted as the relative attenuation of the infected virus to the unmanipulated/gene-modified host cells (Figure 4b). Thus, relative viral attenuation might help the host immunity to control the virus [83].

## 8. Gene Therapy Strategies against HIV

The Berlin patient, an HIV-positive male United States citizen who was diagnosed with HIV while attending university in Berlin and later suffered from acute myelogenous leukemia, received a transplantation of allogeneic hematopoietic stem cells homozygous for CCR5-Δ32. This resulted in a subsequent functional HIV cure [71,72]. Because CCR5 is critical in HIV infection and transmission, as observed with CCR5-Δ32 homozygous cells resistant to HIV infection [84], the manipulation of CCR5 expression on HIV target cells has been intensively investigated and is considered to be effective [34,35,37,39,40,85,86,87,88,89,90,91,92,93,94,95,96,97,98,99,100,101,102]. CCR5 can be targeted by zinc finger nucleases [103,104], ribozymes [105], CRISPR/Cas9 methods [106], transcription activator-like effector nucleases [106] and shRNAs [86,107,108]. Among these, several gene therapy methods, including one using lentiviral vector LVsh5/C46 that expresses shRNA against CCR5 and HIV-1 entry inhibitor C46 [109] have been tested in clinical trials [93]. While CCR5 is involved in numerous pathologic states, including inflammatory and infectious diseases [110], a complete knockout of CCR5 can be related to an increased sensitivity to some viral infections [111,112]. Therefore, CCR5 gene editing should only be considered for an HIV cure [110].

The targeting of HIV RNA sequences by ribozymes or RNAs [44,99,113,114,115,116,117,118,119,120,121,122,123] and HIV DNA sequences by the CRISPR/Cas9 system [124] has been investigated and is also considered a major strategy [100,125]. The latter method has recently been of great interest, which is primarily because of its potential for targeting and disrupting integrated HIV DNA sequences to achieve a complete cure. A recent study targeted and inactivated the HIV-1 long terminal repeat (LTR) U3 region in vitro by Cas9 and guide RNAs (gRNAs), with no off-target gene editing to the host cells being detected [126]. Another study also successfully targeted the HIV-1 LTR U3 region using the CRISPR/Cas9 system. However, this study also detected the emergence of escape variant viruses mediated by the error-prone non-homologous end joining (NHEJ) DNA repair following the CRISPR/Cas9 targeting in the host cells [127]. The mutagenesis problem with CRISPR/Cas9 has also been observed in the treatments of other diseases [128] but can be a serious problem when targeting the HIV DNAs because the strategy might require sustained expression of Cas9 and gRNA in the potential HIV target cells, which means a sustained risk of mutagenesis [129]. Therefore, an improved method for disrupting HIV DNA while prohibiting the emergence of replication-competent escape variants caused by the NHEJ repair system might be necessary. Nevertheless, radical approaches can still be tested in cultured cells and animals. For example, a recent study demonstrated that the in vivo gene delivery of multiplex single-gRNAs and *Staphylococcus aureus* Cas9 to transgenic mice bearing HIV DNA using an adeno-associated virus (AAV) vector resulted in an efficient excision of HIV DNA in various tissues and organs [130]. If safety concerns are met, such a gene delivery method can be a powerful tool to achieve the systemic elimination of latent viral reservoirs in hematopoietic cells and nonhematopoietic cells, such as astrocytes [131]. Wang et al. (2018) have written a thorough review of topics regarding the targeting of HIV DNA by CRISPR/Cas9 [124].

An alternative to the CRISPR/Cas9 strategy is the silencing approach, which aims to reduce the production rate of HIV viral particles per integrated HIV DNA copy [68]. Lentiviral gene delivery enables RNA-based gene silencing, including the previously characterized small interfering RNA (siRNA) called PromA [30,132]. PromA is a short RNA sequence specific for the two NF-κB binding sites in the HIV LTR U3 region. While specific mRNA cleavage by post-transcriptional gene silencing is the best-known mechanism for siRNAs, PromA triggers TGS, which is mediated by epigenetic changes, such as DNA methylation and heterochromatin formation [116,133]. In fact, PromA has been shown to induce chromatin compaction in the HIV-1 promoter region [133]. This means that in contrast to methods attempting to eradicate HIV DNA, PromA locks and stabilizes the latently infecting HIV provirus and prevents the reactivation of viral reservoirs from stimuli, such as tissue necrosis factor (Figure 2) [31,134,135]. The efficacy of PromA in suppressing HIV-1 replication in vivo was first demonstrated by an HIV challenge study using humanized NOD/SCID/JAK3^null^ (NOJ) mice transplanted with human peripheral mononuclear cells expressing shPromA [135]. Our recent study to extend the results using NOJ mice engrafted with shPromA-transduced CD34^+^ cells and their derivatives further demonstrated that PromA could be an effective gene therapy for protecting bone marrow CD34^+^ cells and the hematopoietic potential of the host from HIV infection (Figure 3) [26].

Other potential gene therapy methods include the secretion of soluble HIV entry inhibitors [38]; the rescue of hematopoiesis, including myelopoiesis, erythropoiesis and megakaryopoiesis using *c-mpl* [136]; the expression of a chimeric human-simian TRIM5α [137]; the expression of p68 kinase [138]; and the expression of HIV Gag mutants [36].

## 9. Application of Gene Therapy Methods to Immunotherapies

This section sheds lights on a different application of gene therapy methods to fight against HIV. Immunotherapy approaches based on gene therapy methods have been extensively investigated. Chimeric antigen receptor (CAR) T cells are engineered T cells expressing CARs for the recognition and killing of target cells [139,140]. Most typical CARs are engineered to recognize an antigen with a monoclonal antibody-derived extracellular domain that is conjugated to T-cell receptor-derived transmembrane and intracellular domains. Therefore, despite the use of T-cell signaling pathways, such CAR T-cell therapies might be regarded as an enhancement of antibody-based therapies [141,142]. To date, most successful CAR T-cell therapies have been against cancers [143]. For example, the high efficacy of the adoptive transfer of CAR T cells recognizing CD19 has been demonstrated for the treatment of patients with B-cell acute lymphoblastic leukemia [144] and diffuse large B-cell lymphoma [145]. In contrast, CAR T-cell therapies may require the manufacture of autologous CAR T cells for each patient and thus are not yet widely available [145]. Several broadly neutralizing antibodies have been considered for generating CAR T cells against HIV infection [146,147,148]. Despite the shared concern of escape mutations with antibody-based therapies, CAR T cells are MHC-independent and more potent than the administration of neutralizing antibodies so better outcomes can be expected. A further improvement of HIV CAR gene therapy has been tested to make CAR T cells HIV-resistant by the insertion of the HIV CAR gene expression cassette into the CCR5 locus, which results in the disruption of CCR5 [148]. Finally, an adoptive transfer therapy using autologous CD34^+^ cells transduced with lentivirus expressing a CD4-based CAR that is able to bind the HIV envelope protein has been tested in humanized mice infected with HIV-1 [149] and pigtail macaques infected with a SHIV [150].

Another exciting gene transfer-based immunotherapy involves programming the production of specific anti-HIV antibodies [151,152]. Compared with vaccination, passive immunization using a set of broadly neutralizing antibodies is customizable, MHC-independent and provides instant and reliable protection against HIV [153,154]. However, neutralizing antibodies need repeated administration to provide prolonged protection [154]. Thus, the concept of antibody gene transfer is to overcome the limitation of passive immunization, which is only transiently effective [155]. It was demonstrated in a humanized mouse model that antibody gene transfer by intramuscular inoculation of an AAV vector encoding a full-length antibody was able to induce the production of the antibody by muscle cells and confer protection against intravenous HIV-1 challenge [156]. In another study, an adenovirus serotype 5 (Ad5) vector encoding an HIV-1-specific broadly neutralizing antibody PGT121 (Ad5.PGT121) afforded a more rapid and robust antibody response than an AAV encoding PGT121 (AAV1.PGT121) in HIV-1-infected bone marrow-liver-thymus humanized mice [157].

## 10. Biosafety and Bioethics Concerns Regarding the Application of Anti-HIV Gene Therapies to Human Germline Cells for Pregnancy

In the last part of the review, I would like to comment on the recent issue raised against anti-HIV gene therapy. In late 2018, it was reported that a Chinese researcher used the CRISPR/Cas9 technology to create twins bearing CCR5 double knockouts to confer HIV resistance [158]. However, the inheritable gene modification of human germline cells culminating in human pregnancy is currently unacceptable [159]. If applied to germline cells, CRISPR/Cas9 could cause additional inheritable mutations in the host genome DNA [128,160,161,162] and the influence of this is not entirely predictable at this moment. Therefore, such an investigation on human germline cells should be limited to nonclinical (i.e., in vitro) studies. Regarding the targeted gene, it should be emphasized that CCR5 knockout has not been proven to be safe. Even if a small population of people, mostly of Caucasian origin, is living without functional CCR5 alleles, this does not mean that the loss of CCR5 is universally harmless. This is partly because CCR5 has been reported to play important roles in some viral infections [111,112]. Moreover, there is an unexcluded possibility that the lack of CCR5 function among those carrying the CCR5-Δ32/Δ32 double mutations could be compensated by accompanying genetic variations that do not exist in the majority of human populations with the wild-type CCR5 alleles. Regarding the protection from HIV-1 infection, CCR5-knockout individuals are still susceptible to the infection of CXCR4-tropic HIV-1 strains despite these risks. We refer to the statement published in 2017 by an American Society of Human Genetics workgroup regarding human germline genome editing [163].

## 11. Conclusions

The evidence suggests that the HIV infection alters the bone marrow hematopoietic potential of the host. This can lead to impaired CD4^+^ T-cell generation and contributes to the loss of peripheral CD4^+^ T cells and the manifestation of AIDS. Further investigations on the topics discussed in this review will collectively enhance our understanding of the important role that HIV gene therapy can contribute toward an HIV cure. Intracellular immunization gene therapies, including silencing approaches, are expected to confer relative viral attenuation without interfering with the HIV genome and assist cellular immunity to kill HIV-infected cells. This will ultimately lead to better viral control and a functional cure. In light of this, the long-term preservation of bone marrow CD34^+^ cells and hematopoietic potential as well as aviremic states and restored peripheral CD4^+^ T-cell counts may be an appropriate endpoint of future anti-HIV gene therapies. With this regard, our recent in vivo study using humanized mice transplanted with shRNA (shPromA)-transduced CD34^+^ cells and challenged with HIV-1 has been described to show that the anti-HIV intracellular immunization gene therapy can indeed protect bone marrow HSPCs. Although the efficacy and safety of those novel gene therapy strategies need further improvements for the future applications to autologous HSPC transplantation of HIV-infected patients, a combination of a gene therapy and host immunization based on preserved bone marrow hematopoietic potential and relative viral attenuation will give a hope for a functional cure. Furthermore, the recent advances in gene therapy-based immunotherapy approaches against HIV have also been described in this review.
